# HCN Channels—Modulators of Cardiac and Neuronal Excitability

**DOI:** 10.3390/ijms16011429

**Published:** 2015-01-08

**Authors:** Stefan Herrmann, Sabine Schnorr, Andreas Ludwig

**Affiliations:** Institut für Experimentelle und Klinische Pharmakologie und Toxikologie, Friedrich-Alexander-Universität Erlangen-Nürnberg, 91054 Erlangen, Germany; E-Mail: Sabine.Schnorr@pharmakologie.uni-erlangen.de

**Keywords:** HCN channels, cAMP, cardiac hypertrophy, dorsal root ganglion, neuropathy, inflammation, nociception

## Abstract

Hyperpolarization-activated cyclic nucleotide-gated (HCN) channels comprise a family of cation channels activated by hyperpolarized membrane potentials and stimulated by intracellular cyclic nucleotides. The four members of this family, HCN1–4, show distinct biophysical properties which are most evident in the kinetics of activation and deactivation, the sensitivity towards cyclic nucleotides and the modulation by tyrosine phosphorylation. The four isoforms are differentially expressed in various excitable tissues. This review will mainly focus on recent insights into the functional role of the channels apart from their classic role as pacemakers. The importance of HCN channels in the cardiac ventricle and ventricular hypertrophy will be discussed. In addition, their functional significance in the peripheral nervous system and nociception will be examined. The data, which are mainly derived from studies using transgenic mice, suggest that HCN channels contribute significantly to cellular excitability in these tissues. Remarkably, the impact of the channels is clearly more pronounced in pathophysiological states including ventricular hypertrophy as well as neural inflammation and neuropathy suggesting that HCN channels may constitute promising drug targets in the treatment of these conditions. This perspective as well as the current therapeutic use of HCN blockers will also be addressed.

## 1. Introduction

The ion channel family of hyperpolarization-activated cyclic nucleotide-gated (HCN) cation channels comprises four members termed HCN1–4. Upon hyperpolarization, all four isoforms generate a characteristic inward current which shows the typical properties of the conductance coined *I*_f_ in the heart and *I*_h_ in the brain [1,2,3,4]. Not surprisingly, the four HCN isoforms vary in several biophysical properties including the time course of activation and deactivation and the sensitivity towards cAMP (cyclic adenosine monophosphate). In addition, the expression profile of the channels is markedly different and characteristic for each isoform. A hallmark used to differentiate between isoforms is the kinetics of activation. HCN1 is the fastest activating channel with an activation constant τ in the range of 30–300 ms, whereas HCN4 represents the most slowly activating isoform with τ between 300 ms and several s. In contrast, HCN2 and HCN3 display intermediateactivation kinetics. The reported τ values of the isoforms depend on the potential used to activate the channel, recording conditions including temperature and the expression system or developmental state of primary cells used.

## 2. Modulation, Expression and Functional Significance of Hyperpolarization-Activated Cyclic Nucleotide-Gated (HCN) Channels

### 2.1. Modulation by Cyclic Nucleotides, Phosphoinositides and Tyrosine Phosphorylation

A second key difference between the isoforms is the differential modulation of HCN channels by cyclic nucleotides. Intracellular cAMP positively modulates the gating of the HCN2 and HCN4 isoforms resulting in a shift of the activation curve by 15–20 mV in the positive direction. In contrast, HCN1 is only slightly shifted (shift by around +5 mV) and HCN3 is not modulated by cyclic nucleotides. cAMP binds to the cyclic nucleotide-binding domain (CNBD) in the carboxy-terminus of the channels. In the cAMP-unbound state, the CNBD acts as an autoinhibitory domain which constrains gating to more hyperpolarized potentials [5,6]. Binding of cAMP relieves this inhibition and shifts gating to more positive potentials. In HCN1, inhibition by the CNBD is much smaller than in HCN2 resulting in only a small shift of the activation curve in the presence of cAMP [5].

Another factor shifting channel activation towards positive potentials to a similar degree as cyclic nucleotides are phosphoinositides including phosphatidylinositol-4,5-bisphosphate (PIP_2_) [7,8]. The modulation by PIP_2_ is independent of cyclic nucleotides and, in contrast to cAMP, is equally effective in all four isoforms [8,9]. Experiments using the sea urchin HCN channel spHCN indicated that the effect of PIP_2_ arises, at least in part, from stabilization of the activated state of the voltage sensor [10].

An independent mechanism regulating channel activation is tyrosine phosphorylation by Src kinase [11,12,13,14]. Src kinase phosphorylation accelerates activation of HCN2 and HCN4, whereas there are conflicting data if it also leads to a shift in the HCN4 activation curve [12,14]. The tyrosine residue conferring the modulatory action of Src kinase is located in the C-linker of the channel representing the region linking the transmembrane segment 6 (S6) to the CNBD. In contrast to HCN2 and HCN4, HCN1 is not modulated by Src kinase [11].

### 2.2. Expression Profile

The expression profile of the isoforms is markedly different and strongly depends on the tissue and cell type analyzed. In addition, this tissue-specific expression pattern may vary among different species. Recent studies have shown that the expression pattern in a given cell type is often broader than initially thought. A prime example is sinoatrial node cells where HCN4 has been determined as the principal HCN isoform. However, we, and others, could demonstrate a significant expression of HCN1 and also a small amount of HCN2 beneath HCN4 in murine sinoatrial cells at the protein level indicating that the three subunits together contribute to *I*_f_ in these cells [15,16]. HCN2 has long been known to be expressed in cardiac ventricular myocytes [1]. Beyond that finding, expression of HCN4 and low amounts of HCN1 [15] and in addition a functional role of HCN3 in ventricular myocytes [17] has been demonstrated.

The regional expression in the nervous system shows characteristic differences between the four isoforms [18,19]. HCN2 is nearly ubiquitously expressed in the brain, while HCN1 is found in the neocortex, hippocampus and cerebellum. Low levels of HCN3 are found in the hypothalamus, whereas HCN4 is concentrated in the thalamus. The coassembly of various combinations of HCN isoforms has been shown mainly by analyzing extracts prepared from whole brain or selected brain regions [20,21,22,23,24,25]. However, definite evidence that these combinations indeed exist as functional heteromers *in vivo* is lacking.

### 2.3. Overview of HCN Knockout Studies

The role of HCN channels in mammalian physiology has been examined intensively by the generation and analysis of HCN-deficient mice. All four isoforms have been mutated by using global and/or conditional gene deletion approaches. We will first present a brief overview of the published HCN-knockout lines before covering recent work regarding the role of the channels in the cardiac ventricle and dorsal root ganglion cells.

Mice lacking HCN1 globally are viable and do not display an overt phenotype. However, these animals are defective in the learning of fast motor tasks due to a malfunctioning of cerebellar Purkinje cells [26]. HCN1-knockout Purkinje cells show an excessive hyperpolarization in response to negative currents resulting in a strongly reduced firing after silent states. In addition, the forebrain-selective deletion of HCN1 resulted in an unexpected enhancement of spatial learning and memory [27]. It was concluded that HCN1 channels physiologically inhibit long-term potentiation at distal dendrites of hippocampal CA1 pyramidal neurons and thereby regulate the dendritic integration of synaptic inputs. HCN1-knockout animals also display a dysfunction of the sinoatrial node resulting in sinus dysrhythmia, sinus pauses and a reduced cardiac output demonstrating that HCN1 contributes to a stable cardiac rhythm generation [16].

In contrast to the lack of HCN1, global deletion of HCN2 results in a pronounced neuronal phenotype characterized by absence epilepsy, tremor, hypoactivity and ataxia [28]. In addition, knockouts display a slight sinoatrial dysrhythmia. The spontaneous mouse mutation *apathetic* carries an insertion in the coding region of the CNBD of HCN2 resulting in a premature stop codon and lack of the truncated HCN2 protein in the brain [29]. As expected, homozygous apathetic animals are phenotypically identical to HCN2-knockout mice as demonstrated by EEG (electroencephalogram) recordings and motor and gait analyses [28,29,30]. The generation of spike-and-wave discharges in HCN2 knockouts may be related to an enhanced activity of thalamocortical neurons as shown by abnormal burst firing. It was proposed that the observed hyperpolarization of the resting membrane potential of HCN2 knockout thalamocortical neurons blocks inactivation from T-type Ca^2+^ channels and thereby promotes low-threshold burst firing in response to depolarizing inputs.

Mice with a disruption of the HCN3 isoform do not show visible behavioral and physical abnormalities. However, these mutants display an acceleration of the late repolarization phase of the cardiac action potential in epicardial ventricular myocytes demonstrating that HCN3 contributes to the action potential in these cells [17] (see below).

The global knockout of HCN4 leads to embryonic lethality probably due to the strongly decreased heart rate of mutant embryos [31]. The time and tissue-specific disruption of HCN4 in adult animals was undertaken by different groups with, however, surprisingly different findings. Two lines generated in the lab of the authors using tamoxifen-inducible CAG (cytomegalovirus early enhancer/chicken β-actin/β-globin)-Cre [32] or HCN4-KiT (HCN4-Knock-in of cre/ERT2) [33] transgenes as drivers for the HCN4 deletion displayed recurrent sinus pauses, but no bradycardia nor a deficit in the β-adrenergic heart rate modulation. In contrast, disruption of HCN4 by the use of a different floxed HCN4 construct combined with a MerCreMer transgene resulted in pronounced bradycardia and death roughly one week after the induction of gene deletion [34]. The reason for the differences in the phenotype of these mutants is not clear but may be related, at least in part, to a potential impairment of other HCN isoforms beneath HCN4 in the HCN4/MerCreMer line [35]. This assumption is supported by a very recent study using mice with a genetic inhibition of *I*_f_ [36].

## 3. Impact of HCN Channels on Cardiac Ventricular Excitability

It has long been thought that the function of HCN channels in the heart is limited to cardiac cells which display a clear cut spontaneous diastolic depolarization phase as part of their action potential. Therefore, it was commonly accepted that the channels do not play a role in the excitability of adult ventricular myocytes. Under normal conditions, HCN channels are only poorly expressed outside the cardiac pacemaking and conduction system [15,37,38,39]. For example, the amount of total HCN transcript is several times lower (by a factor of 7.5) in the murine ventricular myocardium as compared to pacemaker tissue [15]. Interestingly, this low ventricular expression of the channels changes during cardiac disease. Cerbai and colleagues demonstrated for the first time in the mid 1990s, that *I*_f_ current density and occurrence is increased in hypertrophic rat cardiomyocytes and that this increase is directly related to the severity of myocardial hypertrophy [40,41]. Later on, several rat and human studies showed a similar upregulation of *I*_f_ in cardiomyocytes isolated from individuals suffering from various pathological conditions including cardiac hypertrophy, heart failure due to ischemia or pressure overload and dilated cardiomyopathy [39,42,43,44,45]. Therefore, the upregulation of the “pacemaker current” in non-pacemaking tissue may alter the electrical stability of the ventricular myocardium which may eventually increase the risk of life-threatening arrhythmias.

**Figure 1:**
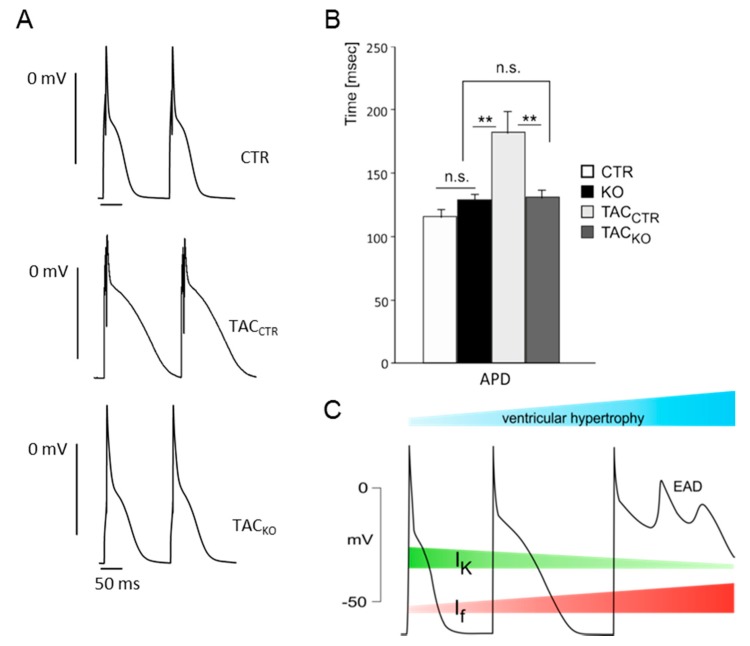
Mice lacking hyperpolarization-activated cyclic nucleotide-gated (HCN) 2+4 channels do not develop a significant prolongation of the action potential during ventricular hypertrophy. (**A**) Action potentials of ventricular myocytes isolated from control (CTR), hypertrophic control (TAC_CTR_) and hypertrophic HCN2+4 knockout (TAC_KO_) mice. Ventricular hypertrophy was induced by the transverse aortic constriction procedure (TAC); (**B**) Mean action potential durations (APD) of ventricular myocytes from controls (CTR, white), HCN2+4 knockouts (KO, black), hypertrophic controls (TAC_CTR_, light grey) and hypertrophic HCN2+4 knockouts (TAC_KO_, dark grey). n.s. means *p* > 0.05, ** *p* < 0.01. (**A**) and (**B**) are taken from Hofmann, F. *et al.* [46]; (**C**) During ventricular hypertrophy, an increased activity of HCN channels, together with a decrease in potassium currents, counteracts the repolarization of the action potential. The resulting action potential prolongation increases the risk of early after depolarizations (EAD) which are an important cause of lethal ventricular arrhythmias. The diagram is partly based on [61]. *I*_f_ and *I*_K_ are indicated by red and green stripes, respectivley. The development of ventricular hypertrophy is indicated by the bluish stripe at the top of the diagram.

Recently we addressed this issue directly by analyzing pro-arrhythmic parameters in *I*_f_ deficient animals before and after induction of cardiac hypertrophy [46]. The complete loss of *I*_f_ in the working myocardium was achieved by the combined deletion of the predominant ventricular isoforms HCN2 and HCN4. By using this conditional double knockout approach, we were able to demonstrate that the increased *I*_f_ in hypertrophic working myocardium alters the repolarization of the ventricular action potential (Figure 1). This was remarkable since, in analogy to sinoatrial node cells, the depolarizing *I*_f_ current should contribute to the (diastolic) depolarization phase of the ventricular action potential rather than repolarization. However, the involvement of *I*_f_ to ventricular repolarization is explainable by the very slow deactivation kinetic of HCN channels. Because of the relatively fast ventricular cycle length a significant fraction of HCN channels remains activated during the entire cardiac action potential [17,47,48,49]. Such HCN background currents are able to counteract ventricular repolarization in phase 2 and 3 of the action potential in the hypertrophied myocardium, where the expression of *I*_f_ current is increased and simultaneously outward potassium currents are reduced. Our ventricular HCN2/4 knockout model identified the enhanced *I*_f_ as an important contributor to the typical electrophysiological alterations observed in the hypertrophic heart including prolonged ventricular action potentials and lengthened QT (time between start of the Q and end of the T wave) intervals [50]. Considering the fact that delayed ventricular repolarization and prolonged QT intervals promote early after-depolarizations and electrical instability, ventricular HCN channels may play a significant role in the diseased heart by increasing the risk for severe ventricular arrhythmias [51,52].

This suggestion is further supported by a recent work of Kuwabara *et al.* who analyzed the effect of ivabradine, an HCN channel blocker, on survival and rhythmicity in a transgenic mouse model of dilated cardiomyopathy [53]. In this study, a dominant negative form of neuron-restrictive silencer factor NRSF (dnNRSF), an important transcriptional repressor of the fetal cardiac gene program, was over-expressed in the cardiomyocytes of transgenic (Tg) animals [54]. In consequence, dnNRSF-Tg mice showed an increased cardiac expression of *I*_f_ and other fetal ion channels and developed progressive cardiomyopathy accompanied by ventricular tachyarrhythmias and sudden arrhythmic death. The survival rate of dnNRSF-Tg animals significantly improved when ivabradine was administrated at low doses which did not affect heart rate. Additionally, in isolated isoproterenol-stimulated transgenic ventricular myocytes ivabradine suppressed spontaneous activity triggered by early after-depolarizations. Interestingly, both the ventricular specific block of HCN channels [53] as well as the ventricular cardiomyoycyte-specific deletion of the HCN2+4 isoforms [46] was without any effect on cardiac structural remodeling and ventricular dysfunction. Hence, the repeatedly reported beneficial effects of ivabradine on left ventricular structural remodeling, cardiac fibrosis, systolic and diastolic function [55,56,57,58,59,60] are probably largely caused by the heart rate lowering mechanism of the drug rather than by a direct ventricular action. In summary, the enhanced expression of ventricular HCN channels due to cardiac disease causes a delayed repolarization of the action potential and directly increases the excitability of the ventricular myocardium.

Remarkably, this concept is not limited to conditions of ventricular hypertrophy where ventricular *I*_f_ is significantly increased. In a thorough analysis of the cardiac phenotype of HCN3 knockout mice, Fenske *et al.* found that the lack of HCN3 enhances the repolarization of epicardial cardiomyocytes, which was the first report of a significant involvement of *I*_f_ channels to ventricular excitability in the adult and healthy heart [17,49].

## 4. Contribution of HCN Channels to the Excitability of Peripheral Neurons

In neurons, *I*_h_ was first discovered by Mayer and Westbrook in a study of mechanisms of anomalous rectification in the embryonic mouse dorsal root ganglion (DRG) [62]. They described the presence of a time- and voltage-dependent conductance activated by membrane hyperpolarization with strikingly similar characteristics to the “pacemaker current” *I*_f_ identified in sinoatrial node cells a few years before [63,64]. Later it was shown that expression of *I*_h_ was most prominent in large and medium sized neurons, whereas only half of small sized DRG neurons displayed a functional *I*_h_ current [65]. Among the different isoforms, HCN1 and HCN2 were predominantly expressed in dorsal root ganglions, while expression of HCN3 and HCN4 was low or undetectable [66,67,68,69]. HCN2 was more frequently found in small sized neurons, whereas HCN1 was the predominant subunit in large neurons [70,71,72,73,74]. These data are consistent with the electrophysiological findings of a faster activating and less cAMP-sensitive *I*_h_ in large and medium sized DRG neurons as compared to the characteristics of the current in small diameter neurons [65,75,76]. The available HCN knockout studies largely confirm this pattern of HCN isoform expression in the different neuronal subtypes of the DRG. The deletion of HCN1 abolished *I*_h_ in most large neurons but left slowly activating cAMP-sensitive *I*_h_, preferably present in smaller DRG neurons unaffected [75]. In contrast, this component was eliminated by (ubiquitous) deletion of HCN2 [77].

Despite the relatively high and broad expression in sensory neurons, the physiological role of *I*_h_ for sensory function is unknown. The results of a huge number of animal studies analyzing physiological sensation *in vivo* by the use of HCN blockers or mouse mutants argue against an obvious function of *I*_h_ in healthy sensory neurons. In addition, the wide clinical use of the *I*_h_ blocker ivabradine for the treatment of stable angina and heart failure is not associated with peripheral sensory side effects such as dysesthesias and paresthesias. It seems that HCN channel function in the peripheral sensory system is limited to pathological pain conditions, which might make these channels an attractive target for the treatment of pain disorders. Several studies reported enhanced HCN channel expression and/or *I*_h_ activity in the peripheral pain pathway following neuronal damage [70,73,78] or inflammation [68,69,74,79].

These observations have raised the hypothesis that nociceptor hyperexcitability, which is associated with hyperalgesia and allodynia, is attributed to an increased activity of *I*_h_ in the pain sensing pathway. This is plausible because HCN channels conduct an excitatory mixed inward current which is typically activated near the neuronal resting membrane potential. A pronounced HCN activity in sensory neurons following nerve injury or inflammation may shift membrane potential to more depolarized values. This lowers the activation threshold of nociceptors and facilitates spontaneous and/or neuronal repetitive activity in line with the increased sensitization to painful stimuli. Such an increased functional importance of *I*_h_ may mechanistically be attributed to enhanced gene expression [68,69,73,74,79,80], PIP_2_ interaction [81], channel phosphorylation [12,82] or to elevated levels of cAMP typically triggered by inflammatory substances such as PGE2 (prostaglandin E2), serotonin and substance P [83,84,85].

### 4.1. Participation of HCN Channels in Neuropathic Pain Behavior

A significant involvement of *I*_h_ to tactile allodynia and thermal hyperalgesia was first demonstrated by studying the analgesic effect of the unselective HCN channel blocker ZD7288 in various neuropathic pain models including the CCI model (chronic constriction injury), the Chung model (L5/6 spinal nerve ligation) and the Seltzer model (partial sciatic nerve injury) [66,70,73,86,87]. In all these models, ZD7288 caused a strong reversal of pain behavior in response to both mechanical and thermal stimulation.

**Figure 2:**
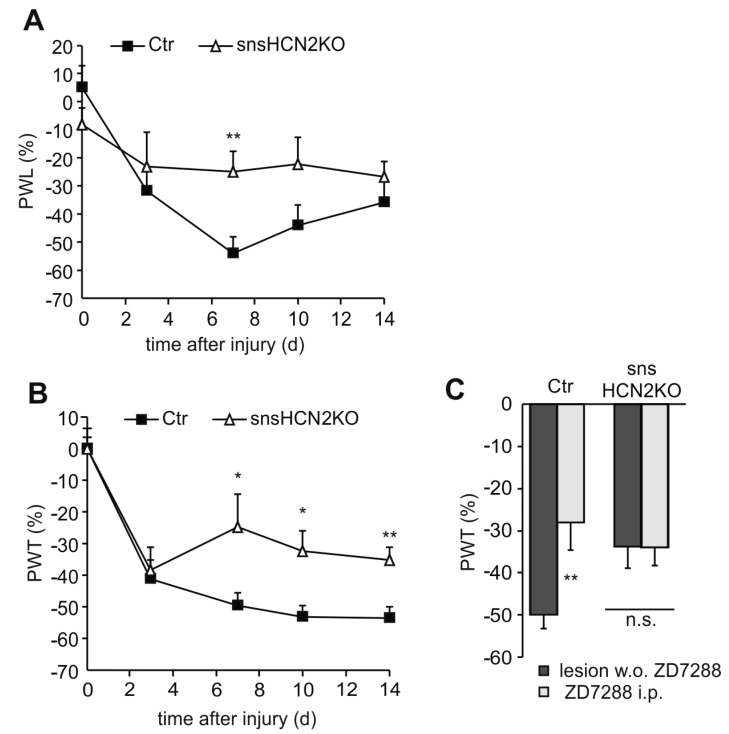
Mice with a selective deletion of HCN2 in peripheral sensory neurons (snsHCN2KO) display reduced neuropathic pain behavior in response to (**A**) noxious heat and (**B**) mechanical stimulation (*n* = 9–10 mice); (**C**) Block of HCN channels by intraperitoneal (i.p.) injection of ZD7288 (4 mg/kg) 14 days after nerve injury significantly decreased mechanical hypersensitivity in controls (Ctr), but had no effect in snsHCN2KO (*n* = 5 mice). All data are presented as relative difference between the injured right and the non-injured left hindpaw ((R − L)/L × 100). PWT and PWL, paw withdrawal threshold and paw withdrawal latency, respectively. * *p* < 0.05, ** *p* < 0.01. n.s., non significant.

HCN channels are widely expressed throughout the peripheral pain pathway. They were found at peripheral and spinal terminals, in the perikaryon as well as along the axon of the DRG neuron [66,68,72,73,74,79,88]. An important site for HCN-mediated hyperexcitability appears to be in the skin and along the axon, since intraplantar as well as perineural administration of ZD7288 significantly reduced neuropathic pain behavior [66,73,86]. It is not clear if presynaptic HCN channels are also involved in the facilitated spinal transmission as a consequence of peripheral nerve injury, because intrathecal administration of ZD7288 produced ambiguous results [70,87] and nonspecific drug effects were suggested [89,90]. Experiments with genetically engineered HCN mutants confirmed the substantial participation of *I*_h_ to neuropathic pain and suggested a pivotal role for HCN2 [74,75,77]. Emery *et al.*, demonstrated that conditional deletion of HCN2 abolished pain behavior in the CCI model [77]. We could largely confirm these results in a different model of neuropathy using a virtually identical HCN2 mutant (snsHCN2KO) [74]. Neuropathy was elicited by unilateral transection of the fourth lumbar spinal nerve innervating the lateral back of the foot and parts of the sole. As compared to baseline, heat hyperalgesia in control mice significantly increased until day 7 and then slowly declined, but still remained elevated (*p* < 0.01, Figure 2A). In contrast, mice with a deletion of HCN2 in nociceptive Na_V_1.8-expressing neurons (snsHCN2KO) developed no significant hyperalgesic behavior as compared to baseline values. Similar findings were observed with mechanical stimulation. After induction of neuropathy, tactile hypersensitivity developed equally for the first three days in controls and knockouts (Figure 2B). However, mechanical withdrawal thresholds then recovered towards baseline in snsHCN2KOs, but not in controls. Furthermore we could exclude a significant participation of HCN2 channels in other cells or other HCN isoforms, since administration of ZD7288 showed no additional effect in snsHCN2KO mice in line with observations made by Emery *et al.* [77].

A peculiar role in neuropathic pain was determined for HCN1. Neuropathic pain behavior in response to heat and pressure was unchanged in HCN1 mutants, but cold allodynia measured with the acetone drop technique was reduced by approximately 50% [75]. This may be relevant for the clinical use of the anticancer agent oxaliplatin. Patients receiving oxaliplatin often suffer from hypersensitivity to cold temperatures. This unpleasant and abnormal sensation may be caused by an oxaliplatin-induced increased HCN1 expression in cold-sensitive small neurons. Consequently, this hypersensitivity was effectively treated in the mouse model by a pharmacological HCN channel block [80].

### 4.2. Participation of HCN Channels in Inflammatory Pain Behavior

A possible involvement of peripheral HCN channels in inflammatory pain conditions has been tested in several animal models using *I*_h_ channel blockers or HCN knockouts, with however surprisingly different results depending on the inflammatory agent and behavioral readout used. A significant analgesic effect of ZD7288 was first seen in the carrageenan inflammation model testing heat hyperalgesia in rats [91]. Later it was shown that HCN2 channels expressed in a subpopulation of small nociceptive neurons were predominantly responsible for this heat hyperalgesia, since the pronounced nociceptive behavior was drastically reduced in conditional HCN2 knockouts after carrageenan or PGE2-mediated inflammation [77]. Interestingly, PGE2-mediated mechanical hypersensitivity was not affected by HCN2 gene deletion or a pan-HCN channel block.

In our laboratory, acute inflammatory states were investigated in snsHCN2KOs after peripheral administration of the membrane permeable cAMP analog 8-bromo-cAMP. The lack of HCN2 in peripheral sensory neurons nearly prevented the development of inflammatory pain behavior in these mice and, in contrast to the PGE2 model, this was evident after both thermal and mechanical stimulation [74]. Hence, peripheral HCN2 channels control the acute phase of neuronal hypersensitivity as long as the inflammatory processes activate signaling transduction pathways, which cause an increase of cAMP in pain-sensing neurons. This fits well with observations made in isolated DRG neurons where cAMP shifted the resting membrane potential to depolarized values and increased action potential firing, which however was not present in neurons isolated from HCN2 knockouts or in the presence of HCN channel blockers [77,92].

On the other hand, this implies that inflammatory compounds which elicit acute neuronal sensitization without elevating cAMP in nociceptive terminals may act via an HCN independent mechanism. This was demonstrated in the zymosan A inflammation model believed to cause hyperexcitability independent of peripheral Gs (G protein α-subunit Gs) signaling [93]. Neither HCN2 deletion [74] nor HCN channel block [94] had any influence on nociceptive behavior in this model.

The concept of increased cAMP levels as a precondition for HCN-mediated sensitization applies to acute inflammatory states, but may not be relevant for chronic inflammatory pain conditions where transcriptional regulatory processes are more important. This fits well to the observed up-regulation of HCN2 protein expression in small DRG neurons and in the spinal dorsal horn after chronic inflammation following peripheral injection of complete Freunds adjuvant (CFA) [68,69,72,74,79]. It was shown, that the enhanced *I*_h_ contributes to neuronal sensitization since deletion of HCN2 as well as administration of ZD7288 reversed tactile hypersensitivity in CFA-inflamed animals [68,74].

The neuronal site of HCN action was studied by analyzing pain behavior in controls and conditional HCN2 mutants (snsHCN2KO) after local administration of ZD7288. The region-specific block at peripheral nociceptors as well as at spinal terminals reduced mechanically evoked pain in controls, but was without any effect in snsHCN2KOs [74]. This suggests that up-regulation of HCN2 lowers the nociceptor threshold as well as facilitates neuronal transmission in the spinal cord. Surprisingly it appears that only tactile hyperexcitability is determined by nociceptive HCN channels, since HCN2 deletion or HCN channel block showed no change in heat hyperalgesia in chronic inflammation models [66,68,74]. These results suggest that mechanical and thermal sensitization are conveyed by distinct neuronal pathways in chronic inflammatory conditions [95,96,97].

## 5. HCN Channel Blockers—Current Therapeutic Use and Perspectives

Ivabradine is the first and to date the only HCN blocker introduced into clinical therapy. In isolated sinoatrial node cells, ivabradine blocks HCN pacemaker currents in the low micromolar range with minimal or no off-target effects. Channel block is accomplished from the cytoplasmic side with a strong preference for the open channel state resulting in use-dependence characteristics. This may minimize the risk for severe bradycardia and improve efficacy during elevated heart rates. At therapeutic concentrations, ivabradine exclusively reduces heart rate without affecting myocardial contractility or intracardiac conduction. Since an elevated heart rate is thought to be an independent cardiovascular risk factor, drugs causing a selective reduction in heart rate should constitute an interesting therapeutic option.

### 5.1. Ivabradine in the Treatment of Coronary Artery Disease

Ivabradine was approved for the treatment of stable angina pectoris in patients with elevated heart rate. It can be used as a single agent when beta-blockers are not tolerated or contraindicated or as add-on therapy when adequate heart rate control is not achieved. It was shown that ivabradine is as effective as the beta-blocker atenolol in its anti-ischemic and anti-anginal efficacy [98]. Two other trials showed that adding ivabradine to an existing beta-blocker therapy provides additional anti-anginal benefits [99,100].

Although ivabradine is effective in reducing angina symptoms, a benefit in terms of prognosis for patients with stable coronary artery disease (CAD) is questionable. The BEAUTIFUL trial investigated such a potential effect of the drug added to standard therapy [101]. This large study (*n* = 10.917) showed that the composite endpoint was not significantly different between the ivabradine and the placebo groups. However, a subgroup analysis suggested a specific benefit for patients with heart rates over 70 bpm [102]. To corroborate this data, a recent study included patients with stable CAD and resting heart rates over 70 bpm (*n* = 19.102) [103]. In the ivabradine group heart rate was reduced to 60 bpm, but unexpectedly there was no reduction in the primary endpoint of cardiovascular death and myocardial infarction. In a subgroup of patients with severe angina, the add-on therapy with ivabradine was even associated with an increased rate of primary endpoints. Taken together, the drug clearly alleviates symptoms of angina, but has not been shown to provide benefits on cardiovascular outcomes in patients with CAD.

### 5.2. Ivabradine in the Treatment of Heart Failure

Ivabradine is recommended for heart failure (HF) patients with reduced left ventricular ejection fraction and elevated heart rate when symptoms are still present despite standard therapy. The SHIFT study included over 6.500 HF patients with increased heart rate and was designed to determine a prognostic effect of ivabradine when added on stable contemporary background therapy [104]. The primary composite endpoint was less frequently observed as compared to placebo (−18%). In particular, the relative risk for hospitalization for worsening HF and deaths caused by HF was reduced by 26%. It was shown that the left ventricular ejection fraction significantly increased and remodeling processes of the ventricle reversed. It appears that most of the beneficial effects are due to the heart rate lowering effect of the drug, but some doubts remain [105]. Our own and other animal studies point to a possible antiarrhythmic effect of ivabradine by blocking ventricular HCN channels, which may contribute to the observed prognostic beneficial effects. Further clinical trials are needed to elucidate if blocking ventricular HCN channels indeed leads to a reduction of ventricular arrhythmia in HF patients.

### 5.3. Isoform-Selective HCN Channel Blockers

A progress in the therapeutic use of HCN channel blockers could be achieved with the development of subtype-selective drugs. Typical side effects of ivabradine are phosphenes (spontaneous visual sensations), which are most probably caused by the block of HCN1 in retinal photoreceptors [106]. A subtype-selective HCN4 blocker should be effective in lowering heart rate without these visual side effects. A selective HCN2 blocker might also be promising, particularly for the treatment of chronic pain conditions including neuropathy and inflammation. As discussed above, HCN2 plays a predominant role in peripheral neuronal sensitization and the development of chronic pain. This is of particular interest since the available therapies for chronic pain states are often unsatisfactory. A compound with high selectivity for HCN2 which does not pass the blood brain barrier may result in an effective pain therapy without cardiovascular or central-nervous side effects.
